# Conducting Polymer–Nanomaterial Hybrids for Cancer Diagnostics

**DOI:** 10.3390/bios16090479

**Published:** 2026-09-01

**Authors:** Mingyu Bae, Jin-Ho Lee

**Affiliations:** 1 Department of Information Convergence Engineering, Pusan National University, Yangsan 50612, Republic of Korea; mgbae@pusan.ac.kr; 2 School of Biomedical Convergence Engineering, Pusan National University, 49 Busandaehak-ro, Yangsan 50612, Republic of Korea; 3 Research Institute of Convergence of Biomedical Science and Technology, Pusan National University of Yangsan Hospital, Yangsan 50612, Republic of Korea

**Keywords:** cancer, biosensor, conducting polymer, nanomaterial, conducting polymer–nanomaterial hybrids

## Abstract

Cancer continues to pose a major global burden because of the high incidence and mortality, underscoring the urgent need for innovative and highly sensitive diagnostic technologies. Conducting polymer–nanomaterial (CP–NM) hybrid biosensors have become promising platforms for cancer biomarker detection, integrating the redox-active and biocompatible nature of conducting polymers such as polyaniline (PANI), polypyrrole (PPy), and poly(3,4-ethylenedioxythiophene) (PEDOT) with the high surface area and charge transport properties of nanomaterials, including metallic nanoparticles, metal oxides, carbon-based nanostructures, and two-dimensional materials. The synergistic interfaces in these hybrids enable efficient electron transfer, signal amplification, and stable biomolecular immobilization, facilitating ultrasensitive and multiplexed detection of proteins, nucleic acids, and metabolites associated with tumor progression. This review highlights recent advances in CP–NM hybrid biosensors for cancer diagnostics, focusing on material design strategies, sensing mechanisms, and representative applications across electrochemical, optical, and mechanical modalities. Finally, key challenges and future perspectives are discussed, emphasizing the potential of CP–NM hybrid platforms to drive next-generation approaches for early cancer detection, therapeutic monitoring, and personalized healthcare.

## 1. Introduction

Cancer remains one of the leading causes of mortality worldwide, representing a major global health burden with millions of new cases and deaths reported annually [1,2]. Although therapeutic modalities have advanced considerably, patients diagnosed at an advanced stage still have limited treatment options and poor clinical outcomes [3,4,5]. Early detection and accurate diagnostics are therefore crucial for enabling timely intervention, guiding therapeutic decisions, and improving overall survival rates [6,7]. Traditional diagnostic approaches, including tissue biopsy, immunohistochemistry, and advanced imaging modalities, have long been indispensable in clinical cancer management [8,9]. However, these methods are often invasive, costly, and time-consuming, with limited ability to detect low-abundance or early-stage tumors [10]. Moreover, spatial and temporal tumor heterogeneity can lead to sampling bias in tissue-based assays, underscoring the urgent need for minimally invasive, highly sensitive, and dynamic diagnostic alternatives capable of capturing real-time molecular changes in cancer progression [11,12].

In recent years, liquid biopsy has become a promising diagnostic approach, offering a noninvasive, rapid, and reproducible method for cancer detection and monitoring [13,14]. By analyzing circulating biomarkers directly from patient biofluids such as blood, urine, or saliva, liquid biopsy enables real-time molecular profiling of tumors without the need for invasive procedures [15]. These circulating biomarkers encompass a diverse array of analytes, including oncoproteins [16,17], nucleic acids [18,19], cytokines and metabolites [20,21,22,23], etc. Each biomarker type provides complementary diagnostic and prognostic information, collectively enabling early-stage cancer detection, therapy response assessment, and relapse prediction [24]. However, realizing the full clinical potential of liquid biopsy demands biosensing technologies with exceptional sensitivity, selectivity, and reliability under complex physiological conditions [25].

Among the diverse materials explored for biosensor development, conducting polymers (CPs) have attracted significant attention for offering electronic conductivity, tunable chemical functionality, and biocompatibility [26]. Their facile processability further enables efficient interfacing with biomolecules and biological fluids [27]. Well-known CPs such as Polyaniline (PANI), polypyrrole (PPy), and poly(3,4-ethylenedioxythiophene) (PEDOT) are the most extensively studied CPs, serving as versatile scaffolds for transducing biochemical interactions into measurable electrical or optical signals [28]. Their conjugated π-electron backbones facilitate charge transport, while their surface chemistry allows for efficient immobilization of biomolecular probes such as antibodies, nucleic acids, or aptamers [29,30]. Furthermore, CPs can be easily fabricated into diverse architectures, including thin films [31,32], nanofibers [33,34], hydrogels [35,36], and porous networks [37,38], allowing integration into miniaturized biosensing devices [39,40].

Despite these attractive features, pristine CPs face intrinsic limitations that can restrict their clinical performance [41]. Pristine CPs often exhibit relatively low surface area, suboptimal conductivity compared to inorganic nanostructures, and susceptibility to chemical or mechanical degradation in physiological environments [42,43]. To address these challenges, CPs are increasingly hybridized with functional nanomaterials such as metallic nanoparticles, metal oxides, carbon-based nanostructures, and emerging two-dimensional materials [44]. In these hybrids, the conducting polymer provides a redox-active, biocompatible matrix for probe immobilization, while the nanomaterial component contributes high surface area and improved charge transport [45]. The resulting composite materials exhibit enhanced signal amplification, sensitivity, selectivity, and operational stability, enabling detection at extremely low concentrations and supporting diverse biosensing modalities for cancer diagnostics.

**Figure 1:**
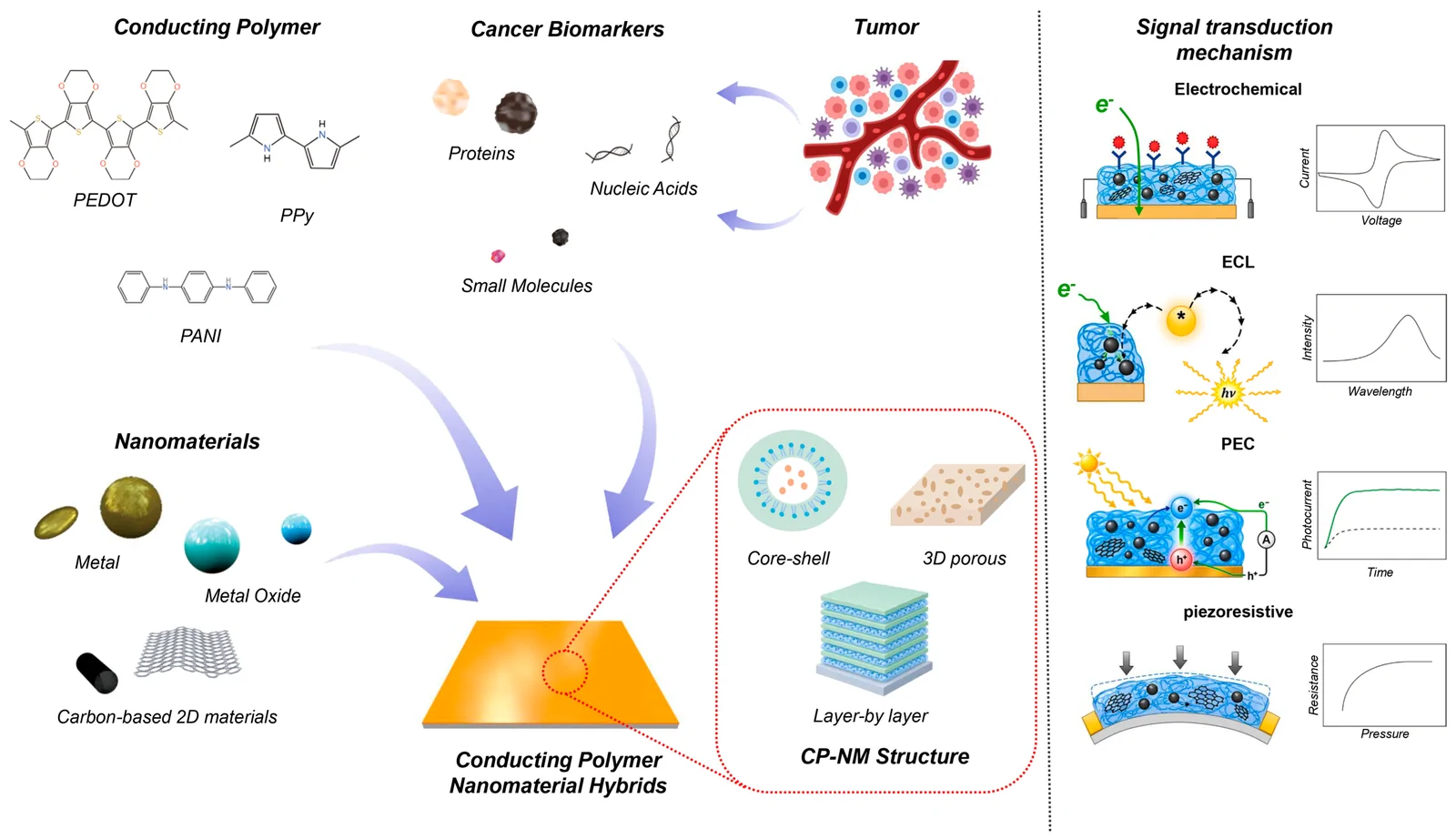
Schematic diagram of development of biosensor via utilization of conducting polymer–nanomaterial hybrids for cancer biomarker diagnostics. Figure created using elements from Servier Medical Art, licensed under CC BY 4.0.

This review provides an updated perspective on conducting polymer–nanomaterial (CP–NM) hybrids for cancer biosensing (Figure 1). We begin by discussing the fundamental electronic, structural, and chemical properties of conducting polymers and highlighting how their integration with nanomaterials enables synergistic functionalities. Subsequent sections focus on the major classes of cancer biomarkers, including proteins, nucleic acids, and small molecules or metabolites. We then present representative examples of diverse detection strategies that exploit CP–NM hybrids to achieve enhanced signal sensitivity and selectivity. Finally, we address current challenges such as reproducibility, long-term stability, and scalability, and we discuss future directions for clinical translation, particularly the integration of these systems with flexible or wearable platforms and artificial intelligence–driven data analytics. In particular, this review goes beyond a study-by-study summary by integrating the discussion of CP–NM composition and architecture with their roles in interfacial charge transfer, biomolecule immobilization, sensing mechanisms, and analytical performance. Representative systems across proteins, nucleic acids, and small molecules/metabolites are further compared to illustrate how differences in material and interfacial design contribute to cancer biosensing performance. Through this integrated analysis, we aim to clarify the fundamental design principles of CP–NM hybrid biosensors, identify key challenges for their further development and clinical translation, and highlight emerging opportunities for real-time and personalized cancer diagnostics and therapeutic monitoring.

## 2. Fundamentals and Design Strategies for Conducting Polymer-Nanomaterial Hybrids

### 2.1. Conducting Polymers: Properties, Advantages, and Limitations

CPs represent a class of organic materials with properties intermediate between conventional insulating polymers and inorganic conductors [46]. Their conjugated π-electron backbones enable charge delocalization, providing intrinsic electrical conductivity while preserving the mechanical flexibility, lightweight nature, and processability characteristic of polymers [29]. This synergistic integration of electrical and mechanical properties makes CPs particularly attractive for biosensing applications, as they can simultaneously provide a biocompatible interface and an active transduction pathway for electronic or optical signal conversion [47]. In particular, CPs are capable of directly translating biochemical recognition events into measurable signals, enabling label-free detection with high fidelity [48]. Moreover, their abundant functional groups facilitate covalent or non-covalent immobilization of biomolecules, improving specificity and stability [30]. The reversible redox activity of CPs further supports dynamic electron exchange with analytes, allowing real-time monitoring of molecular interactions with high sensitivity [49]. Beyond their functional versatility, CPs are compatible with scalable and cost-effective fabrication methods such as spin coating, drop casting, and electro-polymerization, and can be tailored into diverse nanostructures—including thin films, nanofibers, and hydrogels—to suit various biosensing platforms [42].

Among the most extensively studied CPs are polyaniline (PANI), polypyrrole (PPy), and poly(3,4-ethylenedioxythiophene) (PEDOT) [28]. PANI is valued for its environmental stability and reversible protonation/deprotonation redox switching, which facilitates sensitive electrochemical signal modulation in response to analyte binding [50,51]. Its relatively simple synthesis, low cost, and tunable conductivity through protonic doping further support its widespread use in electrochemical sensing platforms [52]. However, its poor solubility and limited processability remain major drawbacks because of the rigid conjugated backbone and strong interchain interactions [53,54]. PPy offers good electrical conductivity, ease of synthesis, and tunable surface properties, making it a widely used scaffold in electrochemical sensors [55,56]. Its biocompatibility and facile electrochemical polymerization also enable direct formation of functional films on electrode surfaces, which is advantageous for biosensor fabrication [57]. Nevertheless, PPy is susceptible to irreversible overoxidation and limited cycling stability, which can result in conductivity loss and structural degradation [58]. PEDOT, particularly in the PEDOT:PSS form, combines high conductivity, low bandgap, mechanical robustness, and aqueous processability, rendering it suitable for flexible and wearable devices [59,60]. Its high optical transparency, good film-forming capability, and mechanical flexibility further facilitate the fabrication of thin, conformable sensing interfaces [61,62,63]. Its biocompatibility, non-toxicity, and ability to support cell proliferation have further expanded its applications in biomedical and biosensing platforms, while the tunable surface chemistry allows efficient functionalization for selective analyte detection [64,65].

However, despite these favorable properties, CPs still face certain limitations that affect their performance in biosensing applications [41]. CPs generally show lower intrinsic conductivity than that of metallic or carbon-based nanomaterials, which can constrain electron transfer rates and limit signal amplification [66]. The relatively low surface area of pristine CPs restricts biomolecule loading, reducing sensitivity in applications targeting low-abundance analytes [67]. Long-term chemical and mechanical stability under physiological conditions may also be compromised, potentially affecting sensor reliability and reproducibility [42,68]. The key characteristics, strengths, and limitations of representative conducting polymers are comparatively summarized in Table 1.

### 2.2. Hybridization Strategies with Nanomaterials and Synergistic Effects

Nanomaterials have been widely employed in biosensing applications owing to high electrical conductivity, catalytic activity, and large surface area, which collectively enhance detection sensitivity and reaction kinetics [69]. They include various categories such as metallic nanoparticles, metal oxides, carbon-based nanostructures, and two-dimensional (2D) materials [70]. Each of these materials often possesses unique physicochemical and morphological properties, necessitating careful selection for specific applications.

To further leverage these advantages, the integration of nanomaterials with conducting polymers (CPs) has become a transformative strategy to resolve the inherent limitations of CPs and enhance biosensor performance [71,72]. When hybridized with CPs, these nanomaterials complement the electronic, mechanical, and biocompatible properties of CPs, resulting in multifunctional platforms capable of sensitive, selective, and robust biomolecular detection. The synergistic behavior of CP–NM hybrids originates from physicochemical interactions formed at the polymer–nanomaterial interface. The conjugated π-electron backbone of CPs provides charge-transport pathways, while conductive nanomaterials can facilitate interfacial electron transfer and reduce charge-transfer resistance. In carbon-based hybrids, π–π interactions between graphene or CNTs and the conjugated polymer backbone promote electronic coupling and interfacial contact. Hydrogen bonding and electrostatic interactions between functional groups on CPs and nanomaterial surfaces can further stabilize the hybrid structure and influence biomolecule immobilization. In addition, appropriate energy-level alignment between the constituent materials can promote charge redistribution and enhance electrochemical or catalytic activity. Therefore, the improved sensing performance of CP–NM hybrids arises not simply from increased surface area, but from the combined effects of interfacial charge transfer, molecular interactions, and nanoscale structural organization. For example, metallic nanoparticles improve electrocatalytic activity, accelerating redox reactions and amplifying electrochemical signals [73], while metal oxides contribute to chemical stability and specific catalytic functions [74]. Carbon-based nanostructures, such as carbon nanotubes and graphene derivatives, can enhance electrical conductivity and provide abundant binding sites for biomolecule immobilization [75]. Beyond their chemical composition, the dimensionality of nanomaterials is an important structural factor influencing the sensing performance of CP–NM hybrids. Zero-dimensional (0D) nanomaterials, such as AuNPs, provide localized active sites that facilitate biomolecule immobilization and signal amplification. In contrast, one-dimensional (1D) nanomaterials, such as CNTs, form continuous conductive pathways that promote efficient electron transport throughout the hybrid network. Two-dimensional (2D) materials, including graphene and MXenes, provide extended planar interfaces and large accessible surface areas, thereby enhancing interfacial charge transfer, biomolecule loading, and interactions with conducting polymers. Additionally, 2D materials offer tunable electrical/optical properties for advanced sensing modalities [45]. Collectively, these enhancements increase sensitivity, reduce detection limits, and improve selectivity for target analytes. Accordingly, 0D, 1D, and 2D nanomaterials contribute to sensing performance through distinct mechanisms involving localized signal amplification, conductive network formation, and extended interfacial interactions, respectively.

To maximize these benefits, various hybridization strategies have been developed to optimize the interface between CPs and nanomaterials (CP-NM) [44,76]. Generally, nanocomposites combine CPs with nanomaterials to improve electron transport, surface area, and mechanical integrity, enhancing sensitivity and response stability [45,77]. Hierarchical architectures such as core–shell structures exploit complementary properties of the core and shell materials to facilitate efficient charge transfer while protecting the CPs from degradation [78]. Layer-by-layer assemblies allow precise control over film composition, thickness, and surface functionality, enabling tailored sensor performance [79]. Three-dimensional porous and flexible frameworks increase accessible surface area for biomolecule binding while maintaining mechanical flexibility [80,81]. Furthermore, CP–NM hybrids can also enable multimodal sensing platforms by combining electrochemical, optical, and mechanical detection modalities in a single device [75,82]. This versatility is critical for cancer diagnostics, where simultaneous detection of multiple biomarker types can improve diagnostic accuracy and facilitate real-time monitoring of disease progression or therapeutic response.

In summary, CP-NM hybrids harness the complementary properties of both materials to overcome individual limitations, amplify sensing performance, and enable multifunctional cancer biosensing platforms. Through careful tailoring of nanomaterial selection, hybridization strategies, and device architectures, these systems could deliver highly sensitive, selective, and stable cancer diagnostics that contribute to next-generation point-of-care devices, real-time monitoring, and personalized medicine. While CPs alone provide a promising foundation owing to their intrinsic electronic and structural features, ongoing research highlights that their full potential in clinical diagnostics is best realized through innovative hybridization with advanced nanomaterials.

## 3. Conducting Polymer-Nanomaterial Hybrids in Cancer Biomarker Detection

A thorough understanding of conducting polymers (CPs) and their hybridization with nanomaterials provides a foundation for designing multifunctional biosensing platforms [44,76]. By combining the electronic conductivity, chemical versatility, and processability of CPs with the high surface area and signal-amplifying properties of nanomaterials, CP–NM hybrids efficiently convert molecular recognition events into measurable signals [45]. This integration enables precise control over charge transport, biomolecule immobilization, and signal transduction, addressing key challenges in detecting low-abundance biomarkers within complex physiological matrices [77]. In cancer diagnostics, such synergy allows sensitive, selective, and robust detection of a wide range of circulating biomarkers, including proteins, nucleic acids, and small molecules/metabolites. The following subsections summarize recent advances, organized by biomarker type and detection modality, highlighting structure–function relationships, detection limits, linear ranges, and clinical relevance.

### 3.1. Protein Biomarkers

Protein biomarkers are among the most clinically relevant indicators of cancer, providing direct information on tumor progression, metastasis, and therapeutic response [83,84]. CP–NM hybrids have been widely explored for protein detection due to their ability to form hierarchical, conductive, and chemically functional interfaces that support efficient electron transfer, stable immobilization of biorecognition elements, and signal amplification [85]. Electrochemical methods dominate this field because of their quantitative precision and compatibility with miniaturized, point-of-care devices, while emerging optical (e.g., fluorescence, electrochemiluminescence) and mechanical (e.g., pressure-based) transduction strategies further expand the versatility of CP–NM platforms [75,80,86,87]. Protein biomarker detection using CP–NM hybrids can be broadly divided into two categories: (1) tumor-associated markers and (2) immune-/cytokine-related markers.

#### 3.1.1. Tumor-Associated Protein Biomarkers

Tumor-associated proteins such as HER2, CA125, AFP, and CEA serve as critical indicators for cancer diagnosis, prognosis, and therapeutic monitoring [88,89]. Early and sensitive detection of these biomarkers is essential for timely intervention and personalized treatment [89]. CP–NM hybrid biosensors have emerged as powerful platforms, combining the complementary advantages of each component to enable ultrasensitive, selective, and rapid biomarker detection.

**Figure 2:**
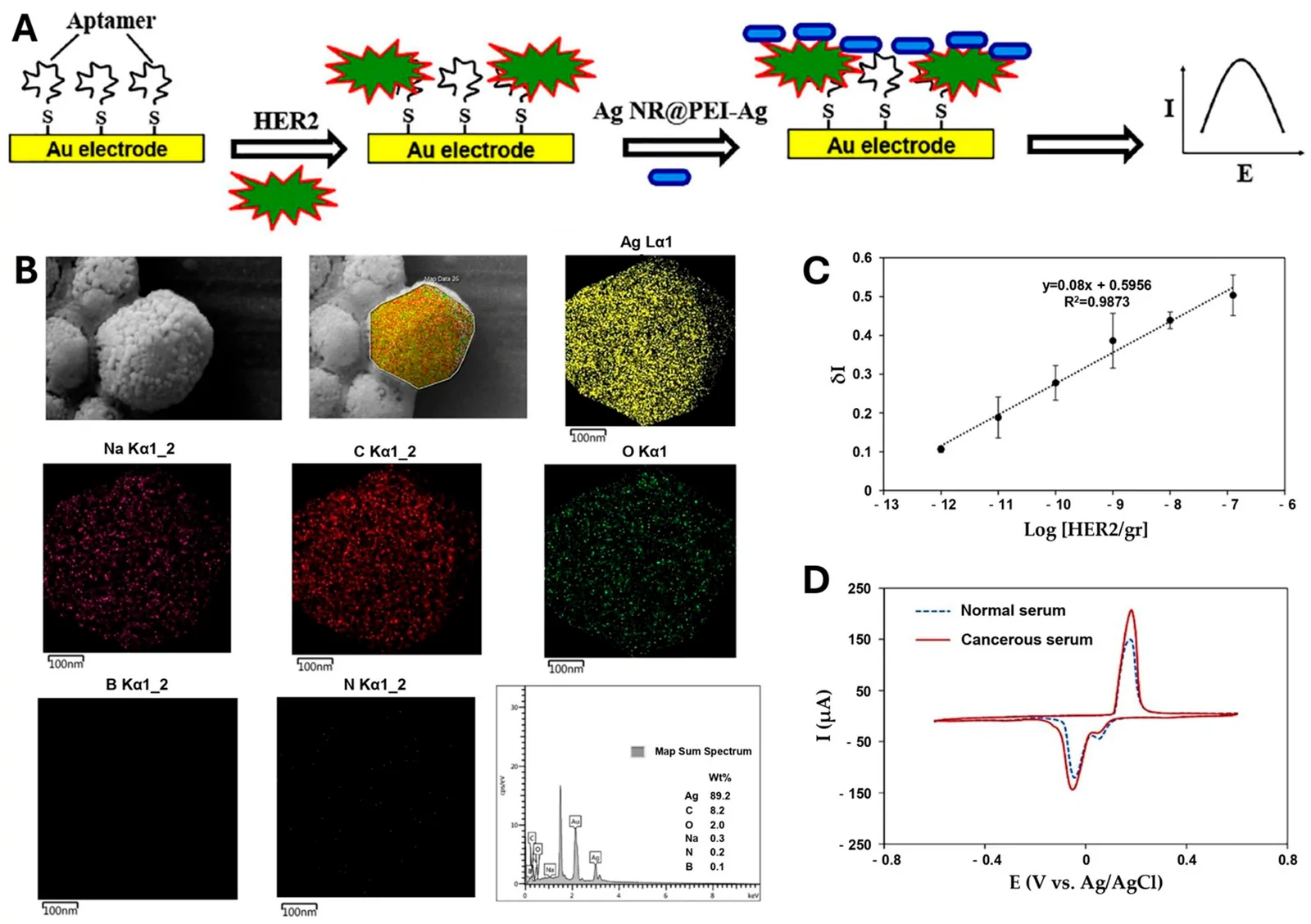
(**A**) Schematic illustration of the electrochemical aptasensor composed of Ag nanorod@polyethylenimine-Ag (Ag NR@PEI-Ag) nanohybrids for HER2. (**B**) SEM image, elemental X-ray mapping and EDX analysis of the as-prepared Ag NR@PEI-Ag nanohybrid. (**C**) Calibration plot for determination of HER2. (**D**) Aptasensor responses to human serum, including cancerous and normal. Reproduced with permission from [91], published by Scientific Reports 2023.

A representative example is HER2, a transmembrane tyrosine kinase receptor overexpressed in aggressive breast and gastric cancers [90]. Hakimian et al. developed an electrochemical aptasensor by integrating Ag nanorods with polyethylenimine-modified Ag shells (Ag NR@PEI-Ag) nanohybrid as a dual-functional signal amplifier [91]. In this architecture, the metallic silver core acts as a redox-active center, while the PEI-modified outer layer enhances electron mobility, surface charge interaction, and aptamer anchoring density. Upon target binding, the positively charged nanohybrids electrostatically adsorbed onto the negatively charged aptamer–HER2 complex, establishing an efficient redox-coupled interfacial network that produced a sharp voltammetric signal. The sensor achieved a linear detection range from 10^−12^ to 10^−7^ g with an LOD of 10 pg, and delivered results in 30 min, exemplifying rapid, noninvasive detection (Figure 2). Extending this signal amplification strategy, Ana L. Soares et al. applied PEDOT nanotubes decorated with Au nanoparticles, achieving ultrasensitive detection of folate-binding protein (FBP), a marker associated with cellular proliferation and cancer metabolism [92]. The conductive PEDOT matrix enhanced electron transfer kinetics, while AuNPs increased the surface area for aptamer immobilization. The resulting hybrid enabled a linear range of 0.0001–0.1 nmol L^−1^, with an LOD of 4.5 pmol L^−1^.

Beyond signal amplification, CP–NM hybrids can also improve probe immobilization density, enabling mediator-free or label-free detection. For instance, Kanagavalli et al. detected Claudin18.2 (CLDN18.2), a tight junction protein overexpressed in gastrointestinal and esophageal cancers, using amine-rich polymelamine (PM) electrografted onto graphene (Gr) and carbon nanotube (CNT) electrodes [93]. The PM layer provided stable redox activity and covalent antibody immobilization, while the carbonaceous network enhanced electron transport and sensor surface area. Differential pulse voltammetry demonstrated excellent selectivity against non-target proteins such as CD1, PDCD1, and ErBb2, achieving LODs as low as 7.9 pg/mL. This mediator-free approach illustrates the potential of CP–NM hybrid immunosensors for rapid, cost-effective early cancer detection.

The CP–NM hybrids have also been successfully applied to ovarian cancer biomarkers such as CA125 [94]. Foroozandeh et al. developed a g-C_3_N_4_/Fe_3_O_4_/PANI nanocomposite to improve mass transport and aptamer immobilization [95]. The electron-rich conjugated framework provided efficient charge transport, Fe_3_O_4_ nanoparticles facilitated mass transport, and the conductive polymer matrix offered abundant functional groups for aptamer immobilization. Using methylene blue as a redox probe, the sensor achieved a broad linear range of 5–60 U·mL^−1^ with an LOD of 0.298 U·mL^−1^, demonstrating excellent selectivity, stability, and the ability to discriminate between normal and patient serum samples. Complementing this electrochemical strategy, Huang et al. illustrated the versatility of CP–NM hybrid strategies by proposing an innovative magneto-controlled microfluidic voltammetric immunoassay [96]. Anti-CA125 antibody-functionalized magnetic beads served as capture probes, while silver–polypyrrole (Ag–PPy) nanohybrids acted as electroactive nanotags in a sandwich immunoreaction. Upon target binding, the magnetic immunocomplexes were driven to the electrode surface, where Ag–PPy nanohybrids amplified the voltammetric signal through their superior conductivity and redox activity. The system achieved a dynamic range of 0.001–300 U·mL^−1^ with an LOD of 7.6 mU·mL^−1^, demonstrating high specificity, reproducibility, long-term stability, and reliable performance in human serum. Additionally, the magneto-controlled interface could be regenerated by detaching and reattaching the external magnet, emphasizing reusability and point-of-care applicability. Further advancing CP–NM hybrid design, molecularly imprinted polymers (MIPs) have been applied to gold screen-printed electrodes (Au-SPE/MIPPy) for dual-mode detection using electrochemical and surface plasmon resonance (SPR) readouts by Rebelo et al. [97]. Electropolymerization of pyrrole in the presence of CA125 as a template generated highly selective recognition sites. Square wave voltammetry enabled quantitative detection with a wide linear range of 0.01–500 U/mL and an ultralow LOD of 0.01 U/mL. The sensor exhibited excellent selectivity against non-target proteins and reliable recovery (91–105%) in artificial serum, demonstrating both sensitivity and analytical applicability in a serum-mimicking matrix.

Along with conventional electrochemical readouts, CP–NM hybrids also enable alternative transduction strategies that highlight their versatility. Mechanical or pressure-based sensing represents one such nontraditional modality, where biochemical interactions are converted into measurable mechanical changes. For instance, Yu et al. developed a portable, pressure-based point-of-care (POC) immunosensor for the sensitive detection of carcinoembryonic antigen (CEA), a widely used biomarker for colorectal, breast, and lung cancers, using a three-dimensional (3D) polypyrrole (PPy) foam–based flexible pressure transducer [80]. In this system, platinum nanoparticles (PtNPs) conjugated to detection antibodies catalyze hydrogen peroxide (H_2_O_2_) decomposition, generating oxygen within a sealed microchamber. The resulting gas pressure increase, proportional to CEA concentration, is transduced by the highly compressible and conductive 3D PPy foam into measurable electrical signals. This hybrid design enables a wide dynamic detection range (0.2–60 ng/mL) with an LOD of 0.13 ng/mL, demonstrating strong reproducibility, specificity, and agreement with ELISA measurements. By converting biochemical recognition into mechanical–electrical signals, this approach exemplifies a novel class of nontraditional transduction platforms for portable, rapid, and quantitative cancer biomarker detection in point-of-care settings.

In a similar context, though with a different mechanism, CP–NM hybrids have been applied in an optical sensing modality. α-fetoprotein (AFP), a key biomarker for liver cancer, was sensitively detected by Wang and colleagues using aptamer-functionalized g-C_3_N_4_/polypyrrole (PPy) nanohybrids through electrochemiluminescence (ECL) sensing [87]. In this design, the graphitic carbon nitride framework promoted efficient electron transfer and enhanced the signal-to-noise ratio, while the conductive PPy polymer improved aptamer immobilization and conformational stability. The synergistic combination of the two materials enabled ultrasensitive detection over a wide linear range of 10^−11^–10^−5^ µg/mL, achieving an exceptionally low LOD of 10^−11^ µg/mL, suitable for early-stage liver cancer monitoring. These results demonstrate how conjugated CP–NM interfaces can optimize both charge transfer dynamics and biorecognition density to enhance analytical sensitivity.

**Figure 3:**
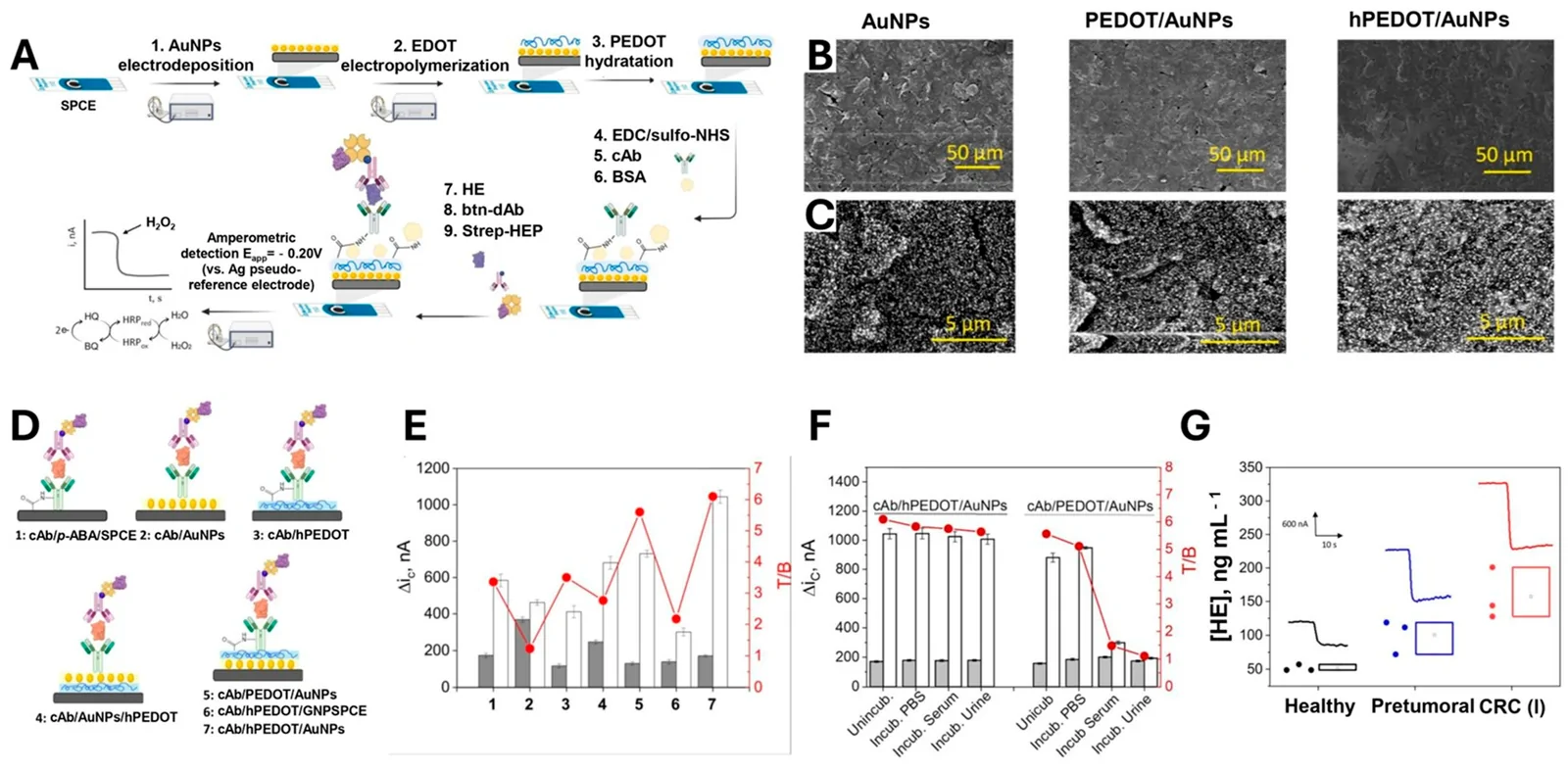
(**A**) Schematic representation of the step-by-step immunoplatform preparation and amperometric transduction using the HRP/H_2_O_2_/HQ system. (**B**) Secondary electron SEM images of SPCEs modified with AuNPs, PEDOT/AuNPs, and hPEDOT/AuNPs. (**C**) Backscattered electron SEM images of SPCEs modified with AuNPs, PEDOT/AuNPs, and hPEDOT/AuNPs. (**D**) Schematic representation of different immunosensing configurations evaluated. (**E**) Evaluation of the recognition efficiency of the different immunoplatforms by comparing the amperometric responses they provided in the absence (B signals, gray bars) and in the presence (T signals, white bars) of 1 ng mL^−1^ HE and the resultant T/B ratio (red dots). (**F**) Evaluation of cAb/hPEDOT/AuNPs/SPCE and cAb/PEDOT/AuNPs/SPCE immunoplatforms’ antifouling behavior. (**G**) HE concentrations in plasma of healthy individuals (black), individuals with premalignant lesions (blue), and CRC patients at stage I (red). Reproduced with permission from [98], published by Elsevier B.V. Licensed under CC-BY-NC 4.0.

Emerging hybrid polymer–hydrogel systems further enhance the clinical applicability of tumor biomarker detection. Human endostatin (HE), an early colorectal cancer (CRC) biomarker, was sensitively detected by Serafín and colleagues using hydrated poly(3,4-ethylenedioxythiophene)–gold nanoparticle (hPEDOT–AuNP) hybrid polymer–hydrogel composites [98]. The synergistic integration of hPEDOT and AuNPs generated a highly conductive, hydrophilic, and biocompatible matrix with superior antifouling and electron transfer properties. The hydrated scaffold improved biomolecule accessibility and stability, while the thiophene–AuNP interaction enabled covalent, oriented antibody immobilization. Implemented as a sandwich-type electrochemical immunosensor on screen-printed carbon electrodes, the platform achieved a low limit of detection (LOD) of 0.04 ng/mL, broad linear range, and excellent reproducibility (Figure 3). Importantly, it distinguished healthy individuals, patients with premalignant lesions, and early-stage CRC cases in plasma samples, demonstrating strong diagnostic potential. This example underscores how multifunctional polymer–hydrogel hybrids can combine high sensitivity, antifouling resistance, and clinical reliability, highlighting their promise for next-generation point-of-care cancer diagnostics. Taken together, these studies show that the contribution of CP–NM hybridization to tumor-associated protein sensing depends strongly on the sensing strategy. Electrochemical aptasensors and immunosensors mainly benefit from enhanced probe immobilization, electron transfer, and electroactive surface area, whereas ECL and pressure-based systems exploit CP–NM structures for optical signal enhancement and mechanical-to-electrical signal conversion, respectively. Hydrated PEDOT–AuNP interfaces further demonstrate that conductivity can be combined with antifouling properties for biomarker detection in complex biological samples. Overall, these studies indicate that the sensing enhancement provided by CP–NM hybridization depends on how the individual components contribute to interfacial electron transfer, probe immobilization, catalytic activity, and accessible surface area. Different hybrid architectures therefore achieve improved analytical performance through distinct combinations of these interfacial functions. The recent research on conducting polymer-nanomaterial hybrids in tumor-associated protein biomarker detection is compared in Table 2.

#### 3.1.2. Immune- and Cytokine-Related Protein Biomarkers

While tumor-associated proteins provide direct insight into cancer presence and progression, the immune system’s response to tumors offers complementary information that is crucial for therapy monitoring and prognostic evaluation [100]. Immune-related proteins, including cytokines, checkpoint molecules, and immunogenic cell death markers, reflect dynamic tumor–host interactions and can signal treatment efficacy, immune evasion, or early relapse [100,101]. Leveraging the same material-focused principles applied to tumor biomarkers, CP–NM hybrid biosensors can be engineered to detect these immune markers with high sensitivity, rapid response, and multiplexing capability, extending their applicability from conventional diagnostics to immune monitoring in personalized cancer therapy.

**Figure 4:**
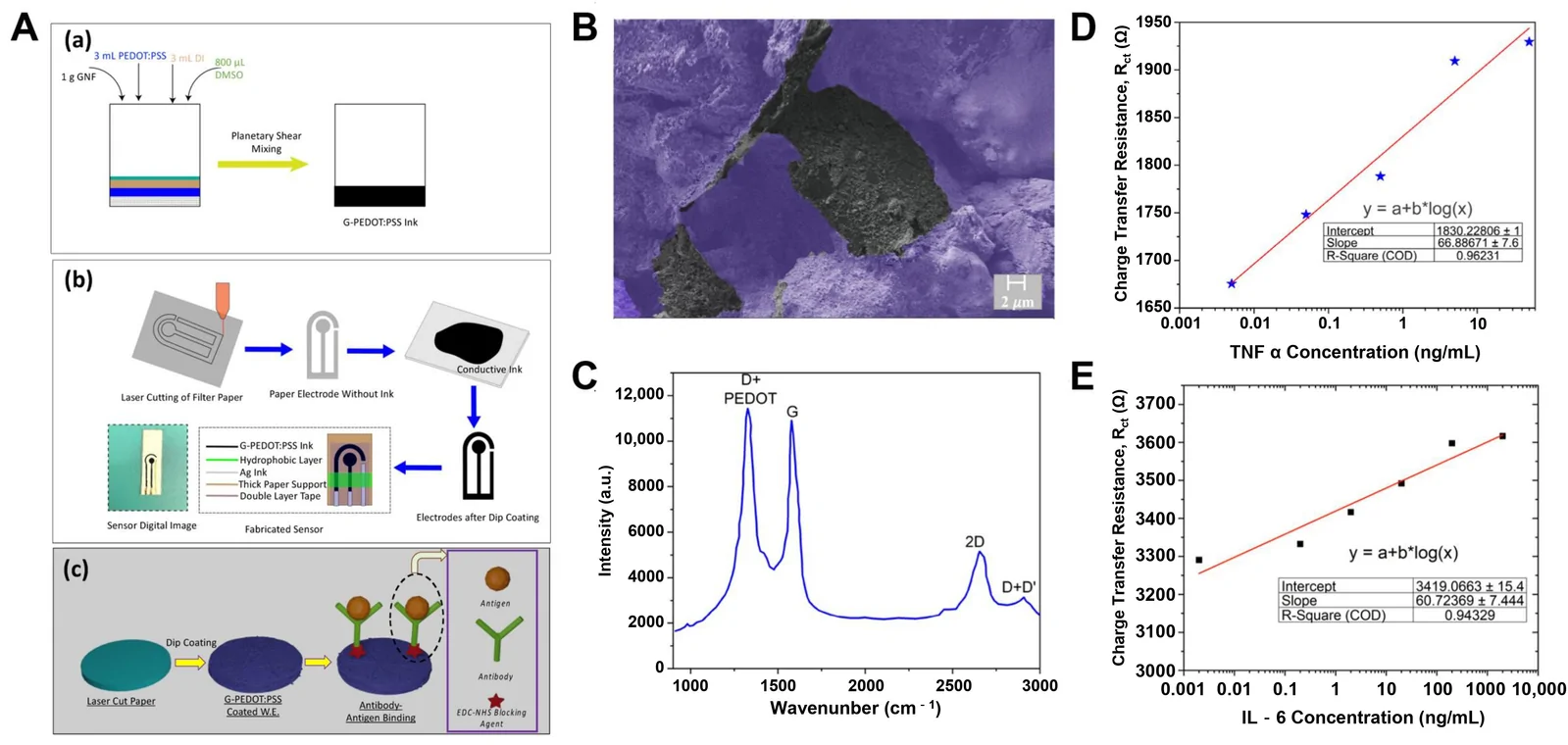
(**A**) Schematic of (**a**) G-PEDOT:PSS conductive polymer ink preparation. (**b**) Paper-based biosensor fabrication. (**c**) Schematic representation of TNF-α/IL-6 detection protocol for paper-based electrodes. (**B**) Cross-sectional SEM images with false color showing graphene attached to the conductive ink and cellulose fibrils at different spatial locations (black indicates the graphene flakes, purple indicates the polymer network). (**C**) Raman microscopy spectrum of the sensor after oxidation. (**D**,**E**) Charge transfer resistance plot with the logarithmic scale of TNF-α and IL-6 concentration. Reproduced with permission from [104], published by Sensors 2023.

Cytokine detection illustrates how these hybrid-material strategies can be applied to immune-related biomarkers. Interferon-gamma (IFN-γ), a key cytokine involved in immune regulation and cancer immunotherapy, serves as a critical biomarker for monitoring patient response to treatment [102]. Saputra et al. developed an ultrasensitive electrochemical aptasensor for IFN-γ detection using a bifunctionalized conducting polymer nanobioconjugate [103]. The sensing probe was constructed by covalently immobilizing IFN-γ-specific aptamers onto a polyterthiophene benzoic acid (pTBA) composite integrated with multi-walled carbon nanotubes and gold nanoparticles (MWCNT/AuNPs). The bifunctional nanobioconjugate incorporated a redox mediator (ferrocene) and an antibody within the polymer backbone, providing efficient electron transfer and highly selective molecular recognition. Under optimized conditions, the sensor achieved an ultrasensitive linear detection range from 0.5 fM to 100 pM, with a detection limit (LOD) of 0.46 ± 0.006 fM (RSD ≤ 5.1%). Moreover, the platform was successfully applied to monitor extracellular IFN-γ release from peripheral blood mononuclear cells and to measure serum IFN-γ levels in patients before and after immunotherapy, where concentrations increased from 0.07 ± 0.004 pM pre-treatment to 0.43 ± 0.02 pM post-treatment. The redox-active polymer scaffold combined with the nanomaterial network enabled rapid electron transfer and selective aptamer recognition, supporting real-time monitoring of IFN-γ in both serum and cell culture supernatants. Building on these strategies, Rahman et al. demonstrated multiplexed cytokine sensing using graphene–PEDOT:PSS-coated paper electrodes, enabling the simultaneous detection of tumor necrosis factor-alpha (TNF-α) and interleukin-6 (IL-6) with high sensitivity (LOD 5.97 pg/mL and 9.55 pg/mL, respectively) and reliable quantification in human serum [104]. The sensing system, a graphene–PEDOT:PSS paper-based electrochemical platform (GCPPS), utilized microliter-scale samples and achieved a wide dynamic detection range of 0.005–50 ng/mL for TNF-α and 0.002–2000 ng/mL for IL-6. Excellent selectivity was confirmed against non-target proteins such as Serpin A1, and the device reliably quantified IL-6 levels in serum with performance comparable to buffer-based assays. Its high-surface-area graphene network combined with the flexible, conductive polymer facilitated rapid electron transfer and stable immobilization of multiple recognition elements, supporting sensitive analysis of immune-related biomarkers in human serum (Figure 4).

Besides cytokines, immunogenic cell death markers offer complementary information by reporting on the intrinsic immunogenicity of tumor cells. Calreticulin (CRT), a multifunctional calcium-binding chaperone, is translocated to the cell surface during immunogenic cell death, functioning as an “eat-me” signal that facilitates phagocytosis and serves as a reliable indicator of cancer cell immunogenicity [105]. In a representative example, Aydın et al. developed a disposable electrochemical impedimetric immunosensor for CRT detection using a nanocomposite of single-walled carbon nanotubes (SWCNTs) and a conducting polymer (PPepx) modified on a screen-printed carbon electrode [106]. The SWCNT–conducting polymer hybrid provided an enlarged surface area and improved electron transfer efficiency, enabling the stable immobilization of anti-CRT antibodies and enhancing sensitivity. The resulting sensor demonstrated a wide linear detection range from 0.015 to 60 pg/mL and an ultralow detection limit of 4.6 fg/mL, outperforming many conventional immunosensors. Importantly, the platform was successfully utilized for human serum samples, distinguishing apoptotic from non-apoptotic conditions. These findings highlight the potential of CP-NM hybrid materials as promising interfaces for real-time, low-cost detection of immunogenic cell death biomarkers and for monitoring cancer therapy response.

Furthermore, immune checkpoint proteins, a key regulator of antitumor immunity, can also be sensitively quantified using CP–NM hybrids. For instance, Pandey et al. developed a nanobody-based electrochemical biosensor to detect soluble cytotoxic T-lymphocyte–associated antigen 4 (sCTLA-4), a serum isoform associated with cancer progression and immune modulation, using a glassy carbon electrode modified with PEDOT:PSS and gold nanoparticles (AuNPs) [107]. Thiolated anti-CTLA-4 nanobodies served as bioreceptors, taking advantage of their high specificity and stability. The conductive polymer scaffold ensured efficient electron transfer, while AuNPs enhanced surface area and signal amplification. The biosensor achieved an ultralow LOD of 1.19 ag/mL over a wide dynamic range (100 ag–500 µg/mL), exhibited excellent reproducibility (RSD < 0.4%) and stability (<3% variation over two weeks), and enabled reliable detection in complex biological samples. This work highlights the versatility of CP–NM hybrids for detecting low-abundance immune checkpoint proteins, providing comprehensive tools for monitoring cancer immunotherapy and patient immune status. Comparison of these approaches shows that CP–NM hybrid design varies according to the target and recognition strategy. The pTBA/MWCNT/AuNP system emphasizes electrochemical signal amplification for IFN-γ detection, whereas the graphene–PEDOT:PSS platform combines a conductive interface with cytokine detection in serum. In contrast, the SWCNT/PPepx and PEDOT:PSS/AuNP architectures integrate conductive nanostructures with specific recognition elements for detecting low-abundance immune-related proteins such as CRT and CTLA-4. Together, these studies demonstrate that rational design of CP–NM hybrid architectures enables highly sensitive and selective detection of immune-related cancer biomarkers across diverse biological samples. Importantly, however, such performance depends not only on signal amplification but also on stable biomolecule immobilization, recognition specificity, and resistance to nonspecific interactions in complex matrices. These considerations become particularly important for multiplexed immune monitoring, where interfacial selectivity and channel reliability must be maintained simultaneously. The recent research on conducting polymer-nanomaterial hybrids in immune- and cytokine-related protein biomarker detection is compared in Table 3.

Beyond the analytical performance demonstrated in these studies, a critical consideration for the clinical application of CP–NM biosensors is their performance in complex physiological matrices. Serum, whole blood, and urine contain abundant proteins, lipids, salts, and other endogenous species that can interfere with target recognition and signal transduction. Non-specific adsorption and biofouling may block recognition sites and alter interfacial charge transfer, whereas cross-reactivity with coexisting biomolecules can compromise analytical selectivity, particularly for low-abundance cancer biomarkers. These limitations can be mitigated through antifouling and hydrophilic interfaces, surface-blocking strategies, selective membranes, sample pretreatment, and optimized recognition elements. Representative studies demonstrate the practical value of these strategies. For example, hydrated PEDOT–AuNP hydrogel interfaces have been shown to provide antifouling characteristics while enabling reliable biomarker discrimination in human plasma [98]. Molecularly imprinted PPy-based sensors have also improved analytical selectivity against non-target proteins and achieved recoveries of 91–105% in artificial serum [97]. Similarly, graphene–PEDOT:PSS platforms demonstrated selective cytokine detection against non-target proteins and maintained reliable analytical performance in human serum [104]. Therefore, the performance of CP–NM biosensors should be assessed not only by sensitivity and LOD under simplified buffer conditions but also by selectivity, recovery, reproducibility, and resistance to matrix-induced interference in complex biological samples.

### 3.2. Nucleic Acid Biomarkers

Nucleic acids, including microRNAs (miRNAs) and oncogenic DNA sequences, play central roles in regulating gene expression, cell proliferation, apoptosis, metastasis, and therapy response, making them highly valuable biomarkers for early cancer detection, prognostic evaluation, and therapeutic monitoring [108]. However, their accurate detection is challenging due to extremely low abundance in biological fluids, vulnerability to enzymatic degradation, and interference from complex biological matrices that can hinder recognition and signal transduction [109]. Conducting polymer–nanomaterial (CP–NM) hybrid platforms have emerged as highly effective strategies for nucleic acid sensing, leveraging the combined advantages of conducting polymers with the high surface area, catalytic properties, and signal amplification of nanomaterials. The synergistic integration of these components enhances probe immobilization, electron transfer kinetics, and target accessibility, enabling rapid, ultrasensitive, and selective detection of miRNAs and oncogenic DNA even at extremely low concentrations.

**Figure 5:**
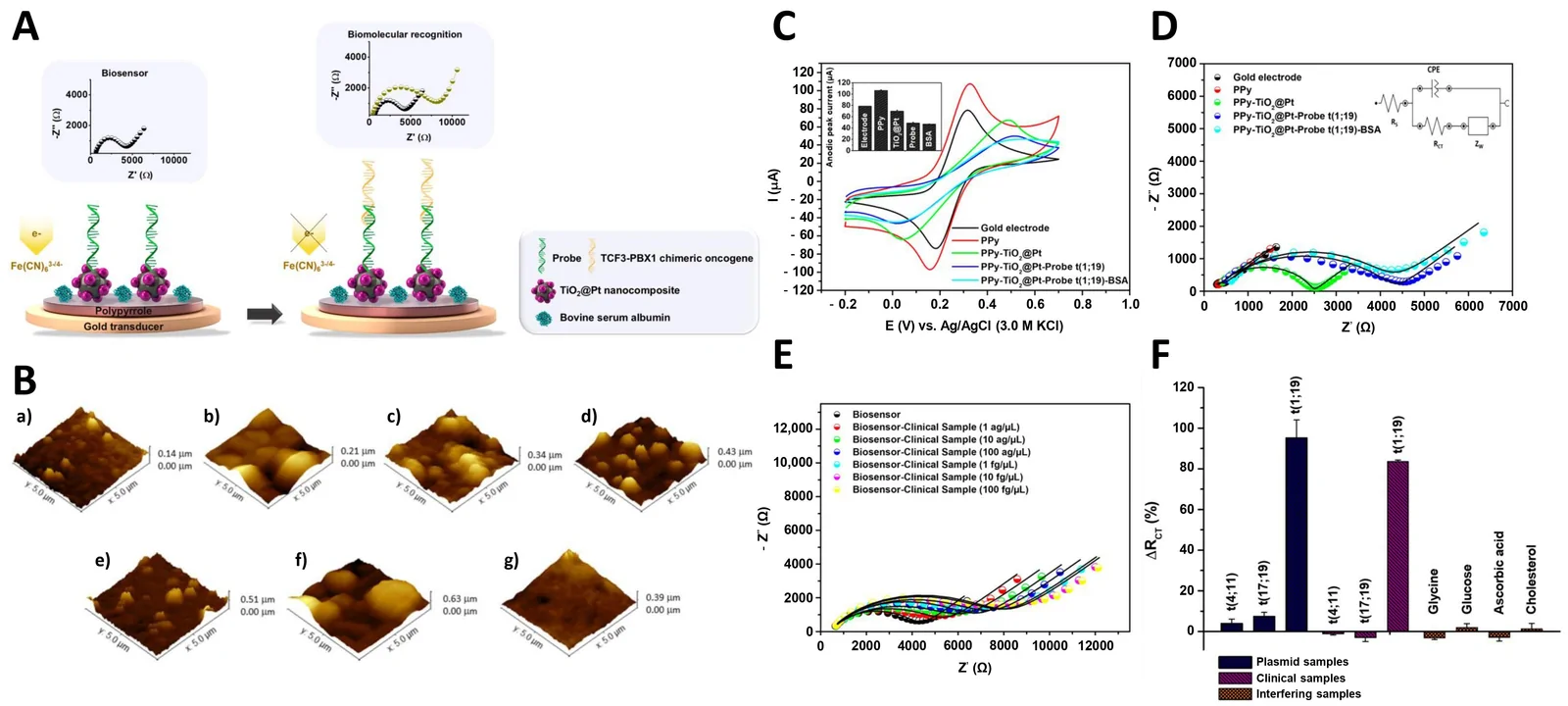
(**A**) Illustration depicting the conceptual stages involved in assembling the electrochemical biosensor. (**B**) AFM images of the following films: PPy (**a**), PPy-TiO_2_@Pt (**b**), PPy-TiO_2_@Pt-Probe (**c**), PPy-TiO_2_@Pt-Probe-BSA (**d**), Biosensor with ALL positive plasmid sample (**e**), Biosensor with ALL positive clinical sample (**f**), and Biosensor with ALL negative sample (**g**). (**C**) Cyclic voltammograms obtained from the characterization of the nanostructured biosensor fabrication steps. Inset: Anodic peak currents recorded during biosensor development and the equivalent circuit used for fitting the spectroscopic measurements. (**D**) Impedance diagrams obtained from the characterization of the nanostructured biosensor fabrication steps. (**E**) Impedance diagrams obtained from the biosensor upon exposure to clinical samples from patients diagnosed with ALL, with concentrations ranging from 1 ag/µL to 100 fg/µL. (**F**) Percentage variation in R_CT_ for the biosensing platform when exposed to recombinant plasmid samples and clinical specimens containing the t(4;11), t(17;19), or t(1;19) translocations. Reproduced with permission from [110], published by MDPI, licensed under CC BY 4.0.

Representative examples highlight the versatility and sensitivity of these hybrid systems across different detection modalities. Electrochemical sensors include the 3D AuNPs/PEDOT platform developed by Ma et al. for miRNA24 detection [81]. The sensor achieved an ultralow limit of detection (LOD) of 0.38 fM across a 1 fM–10 nM range, where the 3D conductive PEDOT network facilitated rapid electron transfer and high-density thiolated probe attachment, while AuNPs further enhanced electron mobility and signal amplification. Thus, demonstrating the capability of CP–NM hybrids to detect low-abundance biomarkers relevant to early-stage cancer. Similarly, Silva et al. reported a polypyrrole/TiO_2_@Pt nanocomposite for the leukemia-associated TCF3-PBX1 fusion gene, attaining an LOD of 19.31 aM across 3.58 aM–357.67 fM [110]. In this sensor design, the TiO_2_@Pt nanocomposite provided a high-surface-area scaffold for probe immobilization and enhanced electron transfer, while the polypyrrole matrix offered conductivity and structural stability, illustrating how the synergy between polymer and nanomaterial components can achieve attomolar sensitivity (Figure 5). Further expanding electrochemical nucleic acid sensing, Zhang et al. developed a label-free electrochemical biosensor based on hybrid polypyrrole/gold nanoelectrocatalysts (ppy@AuNPs) for the ultrasensitive detection of miRNA-21 [111]. This sensor utilized target-induced DNA hybridization to form double-stranded DNA capable of accommodating large amounts of methylene blue (MB) without probe labeling, while the conductive ppy@AuNP nanocomposite served as an efficient nanoelectrocatalyst to accelerate the electrochemical reduction in MB and amplify the analytical signal. The PPy matrix provided excellent electrical conductivity and structural stability, whereas Au nanoparticles increased probe immobilization sites and promoted electron transfer through synergistic electrocatalysis. Consequently, the sensor achieved a broad linear range from 10 fM to 100 nM with a low limit of detection of 5.4 fM, while also exhibiting excellent selectivity toward mismatched miRNA sequences, high reproducibility, and remarkable storage stability. Although these platforms all rely on electrochemical transduction, their signal-enhancement strategies are distinct. The 3D AuNP/PEDOT architecture primarily improves probe loading and charge transport, whereas the PPy/TiO_2_@Pt and PPy@AuNP systems additionally exploit nanomaterial-assisted electrocatalysis to amplify the electrochemical response. This comparison suggests that ultralow nucleic-acid detection can be achieved through different combinations of interfacial area, probe density, and catalytic signal amplification.

Wang et al. also demonstrated photoelectrochemical detection using PEDOT/FeOOH/BiVO_4_ nanohybrids to quantify miRNA-375-3p, achieving an ultralow LOD of 0.3 fM [112]. In this configuration, BiVO_4_ served as a visible-light-absorbing semiconductor generating electron–hole pairs, PEDOT facilitated rapid electron collection and minimized recombination, and FeOOH nanosheets enhanced surface area and stabilized probe immobilization. Hybridization of target miRNAs modulated interfacial charge transfer resistance, converting molecular recognition events into measurable photocurrents, illustrating how light-induced and electrochemical signal transduction can be integrated for heightened sensitivity, rapid response, and excellent reproducibility in complex biological matrices. Optical sensing is also demonstrated by W_5_O_14_/PEDOT-Pt hybrid micromotors reported by Çogal et al., which enabled fluorescent detection of miRNA-21 with a sensitivity of 0.028 nM over 0.1–100 nM [86]. Here, W_5_O_14_ nanosheets provided a high-surface-area scaffold for probe immobilization and catalytic sites, while PEDOT-Pt enhanced conductivity and stabilized DNA probes. The micromotor design actively propelled the hybrids through solution via catalytic fuel decomposition, significantly improving analyte diffusion, mass transport, and collision frequency between targets and immobilized probes. This active motion accelerated hybridization kinetics, reduced assay time, and enabled continuous real-time fluorescent monitoring of molecular interactions, effectively overcoming limitations of passive diffusion in conventional static assays.

Further establishing the exceptional sensitivity of CP–NM hybrids, Soni et al. developed 3D PANI–MoS_2_ hybrid nanostructures for the detection of chronic myelogenous leukemia (CML) DNA [113]. In this configuration, the high conductivity and redox activity of PANI facilitated rapid electron transfer, while the layered MoS_2_ nanosheets provided a large active surface area and abundant binding sites for probe immobilization, collectively enabling efficient charge transport and enhanced hybridization efficiency. Through this system, they achieved an ultralow LOD of 3 × 10^−18^ M across a broad 10^−17^–10^−6^ M range. Building upon these ultralow detection capabilities, Tian et al. demonstrated gold nanoparticle/polypyrrole/graphene (AuNP/PPy/GP) composites for miRNA-21 detection with LODs of 78 aM over broad and biologically relevant concentration ranges [114]. The synergistic combination of conductive PPy, high-surface-area graphene, and plasmonic AuNPs promoted rapid electron kinetics, strong probe anchoring, and signal enhancement, ensuring excellent reproducibility, stability, and analytical precision. The PANI–MoS_2_ and AuNP/PPy/graphene systems further illustrate how dimensionality and multicomponent integration influence nucleic-acid sensing. Layered MoS_2_ provides an extended interface for probe immobilization, whereas the AuNP/PPy/graphene architecture combines a conductive carbon network with polymer-mediated charge transport and nanoparticle-assisted signal enhancement. Together, these studies underscore how structural optimization and nanointerfacing within CP–NM hybrid frameworks can push nucleic acid detection into the attomolar regime, with selected platforms further demonstrating applicability to clinical specimens. Importantly, these findings indicate that such performance is not determined by a single material property, but by the coordinated control of probe accessibility, interfacial charge transport, and signal amplification. Therefore, the rational integration of conductive pathways, accessible recognition interfaces, and signal-amplifying components appears to be a key design consideration for CP–NM platforms targeting nucleic-acid biomarkers. The recent research on conducting polymer-nanomaterial hybrids in nucleic acid biomarker detection is compared in Table 4.

### 3.3. Small Molecules and Metabolites

Small-molecule metabolites and signaling molecules, including reactive oxygen species (ROS), neurotransmitters, and oncometabolites, serve as early indicators of tumorigenesis and metabolic reprogramming [116]. Their detection is challenging due to low physiological concentrations, rapid diffusion, and interference from complex biological matrices such as serum or intracellular environments [117]. Conducting polymer–nanomaterial (CP–NM) hybrid platforms have emerged as versatile tools to address these challenges by facilitating enhanced analyte adsorption, accelerated electron transfer kinetics, and selective recognition, thereby enabling ultrasensitive and selective detection of cancer-relevant small molecules.

**Figure 6:**
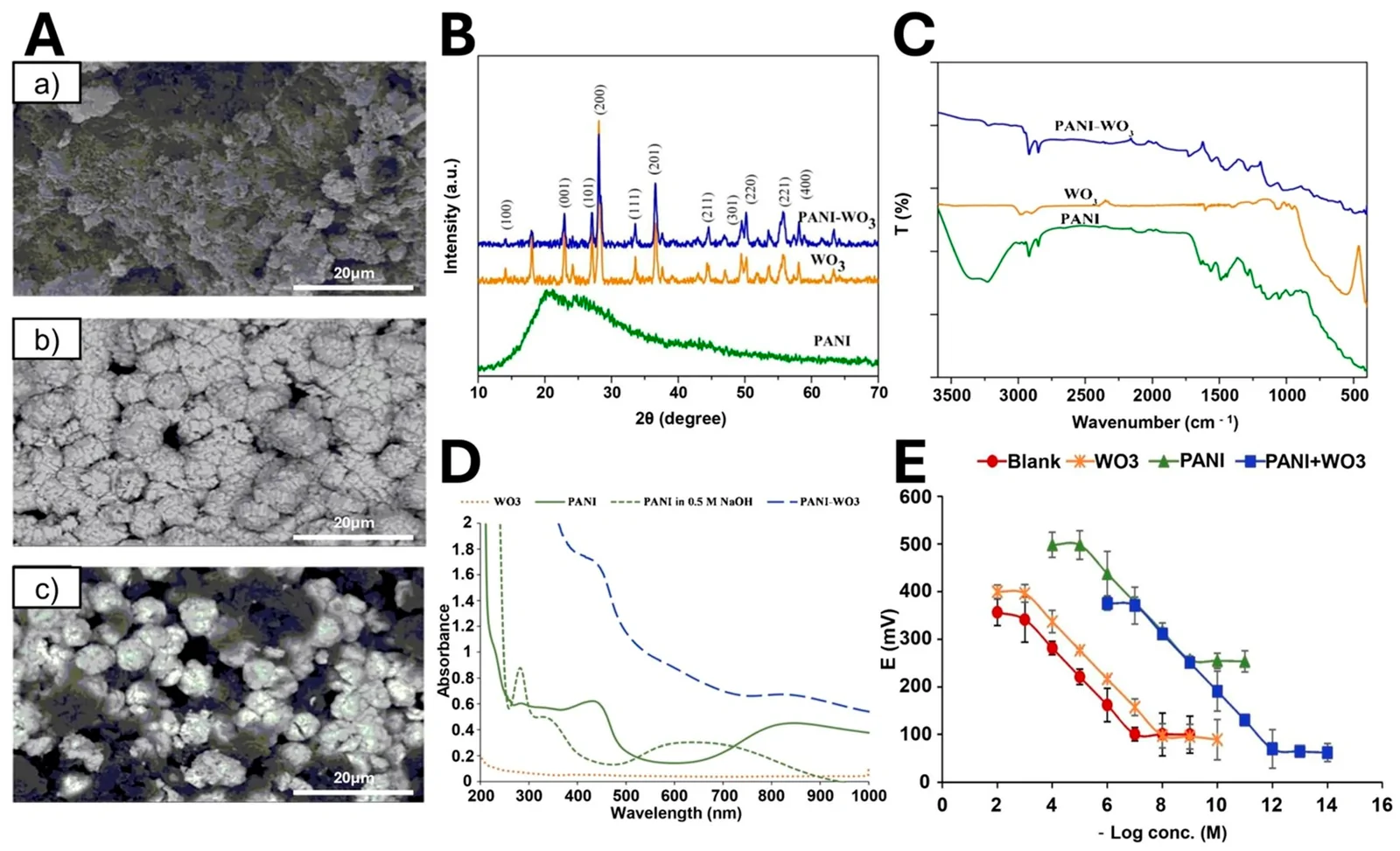
(**A**) SEM images of PANI NPs (**a**), WO_3_ NPs (**b**), and PANI-WO_3_ nanocomposite (**c**). (**B**) XRD patterns of PANI NPs, WO_3_ NPs, and PANI-WO_3_ nanocomposite (**C**) FTIR spectra of PANI NPs, WO_3_ NPs, and PANI-WO_3_ nanocomposite. (**D**) UV-VIS spectra of PANI NPs in the ES form and after dedoping to the EB form using 0.5 M NaOH, WO_3_ NPs, and PANI-WO_3_ nanocomposite. (**E**) Profile of the potential (mV) versus -log concentrations of sarcosine (M) of the proposed sensors. The standard deviation of three measurements (*n* = 3) is represented by the error bars. Reproduced with permission from [120], published by Springer Nature.

Neurotransmitters such as serotonin and dopamine, which are implicated in tumor progression, metastasis, and tumor–immune interactions, have been detected in human plasma and breast cancer cell lines using CP–NM hybrids [118]. For example, Chung et al. developed an electrochemical sensor employing a palladium complex (Pd(C_2_H_4_N_2_S_2_)_2_) anchored on a gold nanoparticle-decorated reduced graphene oxide (AuNPs@rGO) electrode [118]. In this architecture, AuNPs@rGO provides an efficient electron-transfer pathway, whereas the carboxyl-functionalized pTBA layer serves as a stable interface for Pd-complex immobilization, despite partially restricting electron transfer when the polymer layer becomes excessively thick. The subsequent Pd-complex and Nafion layers further improve target discrimination and resistance to interfering species, respectively, illustrating that rational control of individual interfacial layers is critical for achieving selective detection in complex biological samples. This sensor achieved nanomolar-level detection, with limits of 24.0 nM for dopamine and 2.5 nM for serotonin, and dynamic ranges of 0.1–200 µM for dopamine and 0.02–200 µM for serotonin. Notably, serotonin levels in breast cancer cells were approximately 3.1 times higher than in normal cells, demonstrating its ability to distinguish cancer-associated differences in serotonin levels and to monitor tumor-related metabolic changes. Similarly, ROS such as H_2_O_2_, which act as key mediators of oxidative stress, DNA damage, and tumor signaling, have been monitored using a 3D polydimethylsiloxane (PDMS) scaffold coated with PEDOT and functionalized with platinum nanoparticles, developed by Zhang et al. [119]. The 3D conductive network enhanced electron transfer and probe immobilization, achieving sensitive detection in the 0.2–20 μM range with an LOD of 200 nM, enabling dynamic profiling of oxidative stress within tumor microenvironments (Figure 6).

Oncometabolites are particularly important because they reflect tumor metabolic reprogramming, redox balance, and aggressiveness [121]. Sarcosine, a methylated derivative of glycine, is implicated in prostate cancer progression and metastasis by promoting invasiveness and altering cellular methylation patterns [122]. CP–NM electrochemical sensors, such as the polyaniline (PANI)–WO_3_ nanocomposite developed by Yamani et al., have achieved ultralow sarcosine detection limits of 9.95 × 10^−13^ M over a linear range of 10^−12^–10^−7^ M, demonstrating highly sensitive sarcosine detection over a broad low-concentration range [120]. Similarly, glutathione (GSH), a critical intracellular antioxidant that regulates redox homeostasis and drug resistance, can serve as a biomarker for tumor oxidative stress and chemotherapy response [123]. Rajendran Rajaram et al. detected GSH using AuNP-decorated nanoporous PEDOT electrodes, achieving a linear detection range of 0.5–10 μM with an LOD of 0.173 μM, highlighting the ability of CP–NM hybrids to monitor redox-active metabolites at physiologically relevant concentrations [124]. These examples further show that the contribution of CP–NM hybridization depends strongly on the physicochemical characteristics of the target metabolite. While metal-oxide-containing PANI composites can provide catalytically active interfaces for small-molecule oxidation, nanoporous PEDOT/AuNP architectures primarily benefit from high conductivity and increased electroactive surface area. Thus, similar improvements in analytical sensitivity can originate from different material-design strategies. This comparison further suggests that the optimal CP–NM architecture for metabolite sensing should be selected according to the electrochemical behavior and molecular characteristics of the target, rather than relying on a single universally favorable material combination. In particular, balancing catalytic activity, electron-transfer efficiency, and interfacial selectivity is likely to be critical for achieving reliable detection in complex biological environments. These studies demonstrate the versatility of CP–NM electrochemical platforms in detecting low-abundance metabolites that play pivotal roles in cancer biology, offering tools to link metabolic signatures with tumor progression, oxidative stress, and therapeutic response. However, to fully realize the clinical potential of these platforms, future efforts should prioritize improving sensor stability in physiological conditions, minimizing interference from complex biofluids, and enhancing selectivity for structurally similar metabolites, further advancing the clinical utility of CP–NM strategies in cancer diagnostics and therapeutic monitoring. The recent research on conducting polymer-nanomaterial hybrid small-molecule and metabolite biomarker detection is compared in Table 5.

## 4. Conclusions & Future Perspectives

Conducting polymer–nanomaterial (CP–NM) hybrids have emerged as highly versatile platforms for cancer biomarker detection, offering exceptional sensitivity, selectivity, and adaptability across diverse targets, including proteins, nucleic acids, and small molecules or metabolites. By integrating the tunable electronic conductivity, large surface area, and biocompatibility of conducting polymers with the signal-amplifying properties of nanomaterials, these hybrids enable real-time, multiplexed, and point-of-care detection. Rational selection of polymer types, nanomaterials, and hybridization strategies allows the tailoring of sensor performance to specific biomarker classes, thereby supporting personalized cancer diagnostics and therapeutic monitoring.

Despite these advances, several challenges must be addressed for clinical translation. Reproducibility remains a key concern, as variations in polymer synthesis, nanomaterial quality, and hybridization can affect film morphology, surface functionalization, and sensor performance. Long-term stability, antifouling properties, and mechanical robustness are also critical, particularly for wearable and implantable applications. Future efforts should focus on advanced synthesis and assembly strategies to optimize polymer morphology, nanostructure, and electron transfer, while molecular imprinting, redox mediators, and chiral CP composites can further enhance selectivity and sensitivity. Integration with soft materials, hydrogels, and microfluidics will support flexible, stretchable, and multiplexed platforms suitable for point-of-care and real-time monitoring. Despite this potential, reliable multiplexed detection remains challenging because different cancer biomarkers can vary substantially in concentration range, molecular properties, and recognition behavior, making simultaneous detection under a single sensing condition difficult. In addition, the use of multiple recognition elements and signal channels can introduce cross-reactivity, competitive interactions, and channel-to-channel signal variation, which may compromise accurate multianalyte quantification. These limitations become more critical when different transduction mechanisms or signal-amplification strategies are integrated within the same platform. Future CP–NM systems should therefore focus on independently addressable sensing elements, orthogonal recognition and signal-generation strategies, and channel-specific calibration to minimize interchannel interference while maintaining reliable detection across biomarkers with different analytical requirements. As data interpretation is also increasingly important, multimodal CP–NM sensors generate high-dimensional datasets. Coupling sensor outputs with machine learning and artificial intelligence can improve pattern recognition, reduce false positives, and enable predictive diagnostics [125].

Overall, CP–NM hybrid biosensors represent a transformative direction in cancer diagnostics, providing tunable electrical and chemical properties, enhanced signal amplification, and multifunctional detection capabilities. Continued innovation in materials design, hybridization strategies, device engineering, and data analytics will accelerate the development of next-generation point-of-care, wearable, and implantable biosensors, thus enabling personalized, real-time cancer monitoring and guiding adaptive therapeutic strategies.

## Figures and Tables

**Table 1:** Comparison of key characteristics, strengths, and limitations of representative conducting polymers.

Conducting Polymer	Key Characteristics	Strength	Limitation
PANI	Environmental stability, reversible protonation/deprotonation, redox switching	Easy synthesis, Low cost, Tunable conductivity	Poor solubility, Limited processability, Inflexibilit
PPy	Good electrical conductivity, ease of synthesis, tunable surface properties	Biocompatibility, Easy polymerization, High conductivity	Overoxidation, Poor cycling stability, Poor adhesion
PEDOT	High conductivity, low bandgap, biocompatibility, non-toxicity, tunable surface chemistry	High transparency, Good film-forming ability, High flexibility	Hygroscopicity, Acidity, Moisture-induced instability

**Table 2:** Comparison of linear ranges and detection limits for tumor-associated protein biomarker detection.

Conducting Polymer	Hybrid Material	Target	Detection Technique	Linear Range	LOD	Reference
PANI	g-C_3_N_4_/Fe_3_O_4_/PANI nanocomposite	CA 125	Electrochemical	5–60 U/mL	0.298 U/mL	[95]
PPy	ITO/MB-mAb1/CEA/PPy-Ag-pAb2		Electrochemical	0.001–300 U/mL	7.6 mU/mL	[96]
	Au-SPE/MIPPy		Electrochemical	0.01–500 U/mL	0.01 U/mL	[97]
			SPR	0.1–300 U/mL	0.1 U/mL	
	2-NS-PPy/PEE-PPy/2-NS-PPy/AuNP/Apt	CEA	Electrochemical	0.1–1000 ng/mL	0.033 ng/mL	[99]
	PPy foam/Cu/ITO/PET/ Kapton/PDMS/cAb/CEA/ PtNP-labeled dAb		Resistance determination	0.2–60 ng/mL	0.13 ng/mL	[80]
	g-C3N4/PPy/Aptamer	α-Fetoprotein	Electrochemiluminescence	0.01 fg/mL–0.01 ng/mL	0.1 fg/mL	[87]
PEDOT	PEDOT Nanotubes/AuNPs	FBP	Electrochemical	0.0001–0.1 nmol/L	4.5 pmol/L	[92]
	hPEDOT–AuNP hybrid polymer–hydrogel composites	HE	Electrochemical	0.14–1 ng/mL	0.04 ng/mL	[98]
PEI	Ag nanorod/HER2/PEI-Ag nanohybrids	HER2	Electrochemical	0.001–100 ng	10 pg	[91]
PM	SPE/polymelamine-graphene	Claudin18.2	Electrochemical	0.01–100 ng/mL	7.9 pg/mL	[93]

**Table 3:** Comparison of linear ranges and detection limits on immune- and cytokine-related protein biomarker detection.

Conducting Polymer	Hybrid Material	Target	Detection Technique	Linear Range	LOD	Reference
PEDOT	Paper/graphene/PEDOT:PSS	TNF-α	Electrochemical	0.005–50 ng/mL	5.97 pg/mL	[104]
		IL-6	Electrochemical	0.002–2000 ng/mL	9.55 pg/mL	
	AuNPs-PEDOT:PSS/anti CTLA-4 nanobody	CTLA-4	Electrochemical	100 ag/mL–500 µg/mL	1.19 ag/mL	[107]
pTBA	Au nanoparticles layer/pTBA/MWCNT/Aptamer	IFN-γ	Electrochemical	0.5 fM–100 pM	0.46 fM	[103]
PPepx	SWCNTs-PPepx nanocomposite layer	CRT	Electrochemical	0.015–60 pg/mL	4.6 fg/mL	[106]

**Table 4:** Comparison of linear ranges and detection limits on nucleic acid biomarker detection.

Conducting Polymer	Hybrid Material	Target	Detection Technique	Linear Range	LOD	Reference
PANI	3D PANI-MoS2 hybrid nanostructures	CML gene	Electrochemical	0.01 fM–10 µM	3 aM	[113]
PPy	PPy/TiO2/Pt nanocomposite	TCF3-PBX1 gene	Electrochemical	3.58 aM–357.67 fM	19.31 aM	[110]
	PPy@AuNPs/CP-DNA	miRNA-21	Electrochemical	10 fM–100 nM	5.4 fM	[111]
	AuNP/PPy/GP nanocomposite	miRNA-21	Electrochemical	100 aM–1 nM	78 aM	[114]
	AuNPs/graphene/PPy composite	miRNA-21	Electrochemical	1 fM–1 nM	0.020 fM	[115]
PEDOT	3D AuNPs/PEDOT	miRNA-24	Electrochemical	1 fM–10 nM	0.38 fM	[81]
	PEDOT/FeOOH/BiVO4 nanohybrids	miRNA-375-3p	Photoelectrical	1 fM–10 pM	0.3 fM	[112]
	W5O14/PEDOT- Pt hybrid micromotors	miRNA-21	Optical (fluorescent)	0.1–100 nM	0.028 nM	[86]

**Table 5:** Comparison of linear ranges and detection limits on hybrid small-molecule and metabolite biomarker detection.

Conducting Polymer	Hybrid Material	Target	Detection Technique	Linear Range	LOD	Reference
PANI	PANI-WO_3_ nanocomposite	Sarcosine	Electrochemical	1 pM–0.1 µM	0.995 fM	[120]
PEDOT	3D PDMS scaffold/PEDOT/Pt	Reactive oxygen species (H_2_O_2_)	Electrochemical	0.2–20 µM	200 nM	[119]
PEDOT	GCE/Nanoporous PEDOT/AuNP	GSH	Electrochemical	0.5–10 µM	0.173 µM	[124]
pTBA	AuNPs@rGO/pTBA/Pd(C_2_H_4_N_2_S_2_)_2_ complex	Serotonin	Electrochemical	0.02–200 µM	24 nM	[118]

## Data Availability

No new data were created or analyzed in this study. Data sharing is not applicable to this article.
